# Dirac cone intensity asymmetry and surface magnetic field in V-doped and pristine topological insulators generated by synchrotron and laser radiation

**DOI:** 10.1038/s41598-018-24716-1

**Published:** 2018-04-25

**Authors:** A. M. Shikin, A. A. Rybkina, D. A. Estyunin, D. M. Sostina, I. I. Klimovskikh, V. Yu. Voroshnin, A. G. Rybkin, K. A. Kokh, O. E. Tereshchenko, L. Petaccia, G. Di Santo, A. Kimura, P. N. Skirdkov, K. A. Zvezdin, A. K. Zvezdin

**Affiliations:** 1 Saint Petersburg State University, Saint Petersburg, 198504 Russia; 2 Novosibirsk State University, Novosibirsk, 630090 Russia; 3 V.S. Sobolev Institute of Geology and Mineralogy, Novosibirsk, 630090 Russia; 4 A.V. Rzhanov Institute of Semiconductor Physics, Novosibirsk, 630090 Russia; 5 Elettra Sincrotrone Trieste, Trieste, 34149 Italy; 6 Graduate School of Science, Hiroshima University, Higashi-Hiroshima, 739-8526 Japan; 7 Moscow Institute of Physics and Technology, Dolgoprudny, 141700 Russia; 8 A.M. Prokhorov General Physics Institute, Russian Academy of Sciences, Moscow, 119991 Russia; 9 Russian Quantum Center, Skolkovo, 143025 Russia

## Abstract

Effect of magnetization generated by synchrotron or laser radiation in magnetically-doped and pristine topological insulators (TIs) is presented and analyzed using angle-resolved photoemission spectroscopy. It was found that non-equal photoexcitation of the Dirac cone (DC) states with opposite momenta and spin orientation indicated by the asymmetry in photoemission intensity of the DC states is accompanied by the *k*_||_-shift of the DC states relative to the non-spin-polarized conduction band states located at *k*_||_ = 0. We relate the observed *k*_||_-shift to the induced surface in-plane magnetic field and corresponding magnetization due to the spin accumulation. The direction of the DC *k*_||_-shift and its value are changed with photon energy in correlation with variation of the sign and magnitude of the DC states intensity asymmetry. The theoretical estimations describe well the effect and predict the DC *k*_||_-shift values which corroborate the experimental observations. This finding opens new perspectives for effective local magnetization manipulation.

## Introduction

It is well known that 2D metallic-like topological surface states (TSSs) formed at the surface of TIs are characterized by the unique DC helical spin structure with opposite spin orientation for the states with opposite momentum^1–4^. Such TSS spin structure provides a high efficiency for the spin-polarized current generation used in spintronics, for instance, for the spin-current-induced reversal magnetization in ferromagnetic nanoobject contacting with TIs^5–7^. On the other hand, it was shown^8–13^ that the spin-polarized currents along the TSSs can be also generated in TIs by circularly polarized laser radiation due to the asymmetry in photoexcitation for electrons from the branches of the DC states with opposite momentum and spin orientation. In magnetically-doped TIs and 2D-like Rashba systems with helical spin structure (as BiTeI) the photoexcitation by circularly-polarized laser or SR leads to the induced magnetization which can be switched by changing the direction of circular polarization and corresponding asymmetry in the TSS photoexcitation^11,14,15^.

At the same time, a renewed interest is devoted to systems with so called flat magnetism^16–20^ due to their unique magnetic properties and for the perspective use in data storage^21^. Considerable attention is focused on layered magnetic systems with enhanced spin-orbit (SO) coupling based on magnetic TIs, especially when the surface 2D magnetic layer is characterized by the crystalline structure identical to that of TIs used as a substrate, for instance, as in the case of the MnBi_2_Te_4_ quintuple layer arranged on top of GeBi_2_Te_4_ or Bi_2_Se_3_^19,20^. In this case a correlation in crystal structure and atomic composition between the surface layer and the bulk allows to realize the so-called magnetic extension between the DC states of TI and the 2D magnetic layer, allowing to reach an enhanced surface magnetic interaction indicated a large gap open at the Dirac point^19,20^. It significantly differs from the magnetic proximity effect observed for ferromagnetic layer on top of TI^20^.

Magnetically-doped TIs with the surface Curie temperature higher than the bulk one^22^ are very promising in this respect. As done for systems with 2D surface magnetic layer arranged on top a non-magnetic (paramagnetic) bulk with a perfect correlation between the bulk and the surface crystalline parameters, the current work is related to study of similar system - magnetically doped TI at temperatures above the bulk Curie temperature, when only 2D surface ferromagnetism with paramagnetic bulk can be developed in the used temperature region. The bulk Curie temperature for this compound is of about 2.5 K (in accordance with the results of measurements by SQUID and the temperature dependence of the magnetic susceptibility). Therefore, the V-doped TIs, studied in the current work, can be considered at the used temperatures (17–20 K) as materials with paramagnetic bulk and 2D surface magnetic layer. For such kind of magnetic systems we study a possibility of generation of the surface in-plane magnetic field and corresponding magnetization under photoexcitation by synchrotron and laser radiation with varied photon energy at the temperatures above the bulk Curie temperature. We show that the asymmetry in the TSS intensity under photoexcitation by linearly-polarized synchrotron and laser radiation induces an effective magnetic field at the surface both in magnetically-doped and pristine TIs as indicated by the *k*_||_-shift of the Dirac point (DP) position. Both the TSS intensity asymmetry and the resulting DP *k*_||_-shift show a clear dependence on the photon energy. This provide a way for changing the induced in-plane magnetic field and therefore for switching between the spontaneous out-of-plane and the in-plane magnetization in magnetically-doped TI induced by the laser or synchrotron radiation (SR).

## Results and Discussion

**Figure 1:**
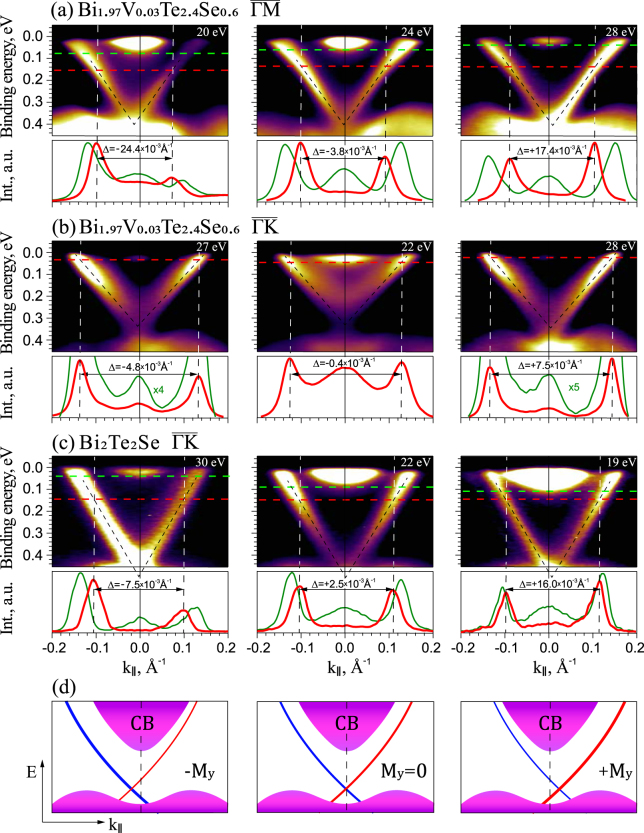
ARPES dispersion maps for V-doped TI with stoichiometry Bi_1.97_V_0.03_Te_2.4_Se_0.6_ measured in the $\overline{{\rm{\Gamma }}{\rm{M}}}$ and $\overline{{\rm{\Gamma }}{\rm{K}}}$ directions (lines (**a**,**b**)) and pristine TI with stoichiometry Bi_2_Te_2_Se in the $\overline{{\rm{\Gamma }}{\rm{K}}}$ direction (line (**c**)), respectively, measured at different photon energies. The reversing of the asymmetry in the intensity of the Dirac cone states with opposite momentum is observed. Below each ARPES dispersion maps – corresponding MDC profiles of the intensity of the DC and bottom CB states measured at the cutting energies marked by the horizontal green and red lines. The cutting energies were chosen for showing the bottom CB states with minimal intensity, i.e. close the CB edge (green lines). (**d**) –Schematic presentation of the *k*_||_-shift of the DC states relative to the non-spin-polarized CB states at different orientation of in-plane magnetic fields (M_*y*_).

Figure 1(a–c) show the experimental ARPES dispersion maps measured at different photon energies for V-doped TI with stoichiometry Bi_1.97_V_0.03_Te_2.4_Se_0.6_ in the $\overline{{\rm{\Gamma }}{\rm{M}}}$ and $\overline{{\rm{\Gamma }}{\rm{K}}}$ directions of the Brillouin Zone (BZ) – lines (a) and (b), and pristine TI with stoichiometry Bi_2_Te_2_Se in the $\overline{{\rm{\Gamma }}{\rm{K}}}$ direction of the BZ – line (c), respectively. The ARPES dispersions were measured at the BaDElPh beamline at Elettra synchrotron. The SR incidence angle was 50° relative to the surface normal when the sample was at normal emission geometry. The ARPES maps demonstrate clearly a pronounced asymmetry in the intensity of the DC states with opposite momenta under photoexcitation by linearly polarized SR both for V-doped and pristine TIs, which varies with photon energy. At certain photon energies a pronounced TSS asymmetry inversion is observed. Below each ARPES dispersion maps the corresponding momentum distribution curves (MDCs), demonstrating the intensity of the DC states with opposite momenta, are presented at the binding energies allowing to show both the states at the bottom of the conduction band (CB) and the DC states. The CB bottom states are used in the current work for estimation of the $\overline{{\rm{\Gamma }}}$ -point position (i.e., *k*_||_ = 0). These states should be symmetrically arranged relative to the $\overline{{\rm{\Gamma }}}$ -point position under the $\overline{{\rm{M}}{\rm{\Gamma }}{\rm{M}}}$ and $\overline{{\rm{K}}{\rm{\Gamma }}{\rm{K}}}$ measurements. In the first approximation, they are non-spin-polarized, and can be a good indicator of the $\overline{{\rm{\Gamma }}}$ -point position. The corresponding MDC profiles are presented at the cutting energies (marked by horizontal green and red lines), where the CB bottom states have minimal intensity and maximally correspond to the $\overline{{\rm{\Gamma }}}$ -point position (blue lines). The presented MDC profiles demonstrate clearly a different intensity of the states at the opposite TSS branches under photoexcitation and a pronounced TSS intensity asymmetry variation with the photon energy. Moreover, one can also see the *k*_||_-shift of the TSS branches relative to the bottom CB states. As we noted before, the bottom CB states should be centered at *k*_||_ = 0 under the $\overline{{\rm{M}}{\rm{\Gamma }}{\rm{M}}}$ and $\overline{{\rm{K}}{\rm{\Gamma }}{\rm{K}}}$ measurements and can be an indicator of the $\overline{{\rm{\Gamma }}}$ -position. In the current work we focus on the observation of the *k*_||_-shift of the spin-polarized DC states (the TSS branches) relative to the non-spin-polarized CB states. As we will show later, this *k*_||_-shift accompanies the variation of the TSS intensity asymmetry in the ARPES dispersion maps versus the photon energy, and can be a direct confirmation of the induced in-plane magnetization and in-plane magnetic field. This field is induced by the hole-generated uncompensated spin accumulation due to non-equal TSS photoexcitation. The positions of the peak maxima of the CB bottom states in each dispersion maps were estimated by fitting of the corresponding MDC profiles with minimal intensity of the CB states, i.e. maximally close to the CB bottom edge. From the presented ARPES dispersion maps one can clearly see the non-symmetric arrangement of the opposite DC branches relative to the CB bottom states. By changing the photon energy the *k*_||_-shift is reverted relative to the bottom CB states in direct relation to the sign inversion in the photoemission TSS intensity asymmetry. This is clearly visible in the profiles of the DC states relative to the bottom CB states. The differences in the *k*_||_-shifts of the DC states with opposite momentum are shown above the arrows in corresponding MDC profiles.

The *k*_||_-shift of the spin-polarized DC states relative to the non-spin-polarized CB states is typically observed under application of an in-plane magnetic field^12,23,24^ in the direction perpendicular to this field. Therefore, the *k*_||_-shift presented in Fig. 1 can be a proper indicator of the SR-induced in-plane effective magnetic field. The correlation between the asymmetry in intensity of the DC states with opposite momentum and the *k*_||_-shift of the DC states relative to the CB bottom states is observed both for the V-doped and for pristine TIs.

Schematic presentation of the *k*_||_-shift of the DC states relative to the bottom CB states at the opposite orientation of the in-plane magnetization (M_*y*_) is shown in Fig. 1(line d). Non-equal photoexcitation of the DC states with opposite momenta is followed by the hole-generated uncompensated in-plane (and out-of-plane) spin accumulation with the spin locked perpendicular to momentum that induces a corresponding in-plane and out-of-plane magnetic field via spin-torque effect^15^. The theoretical estimations of the induced in-plane magnetic field and its relation to the hole-generated uncompensated spin accumulation is presented below. The direction of the induced in-plane magnetic field is aligned parallel to the generated uncompensated spin accumulation (i.e. perpendicular to momentum), which is determined by the asymmetry in the hole generation with opposite momentum. As a result, it is followed by the *k*_||_-shift of the Dirac cone position perpendicular to the direction of the induced magnetic field^12,15,23,24^. By changing the TSS asymmetry sign the induced magnetic field is reversed.

In Fig. 1(a–c) we can see that the asymmetry in the TSS intensity under photoexcitation and the accompanied *k*_||_-shift of the DC branches are inverted at different photon energies, mainly between the regions near *hν* = 20–21 eV and 28 eV. At the same time in the middle of this photon energy range the asymmetry in the TSS intensity is reduced, that is followed by a corresponding reduction of the *k*_||_-shift between the opposite DC states (i.e. the shift of the DP position relative to *k*_||_ = 0). As we noted before, this variation of the DC shift can be an indicator of the orientation variation and the value of the induced in-plane magnetic field with photon energy. This is observed both for magnetically-doped and for pristine TIs under orientation of the SR incidence plane both in the $\overline{{\rm{\Gamma }}{\rm{M}}}$ and $\overline{{\rm{\Gamma }}{\rm{K}}}$ directions, while in different degree.

**Figure 2:**
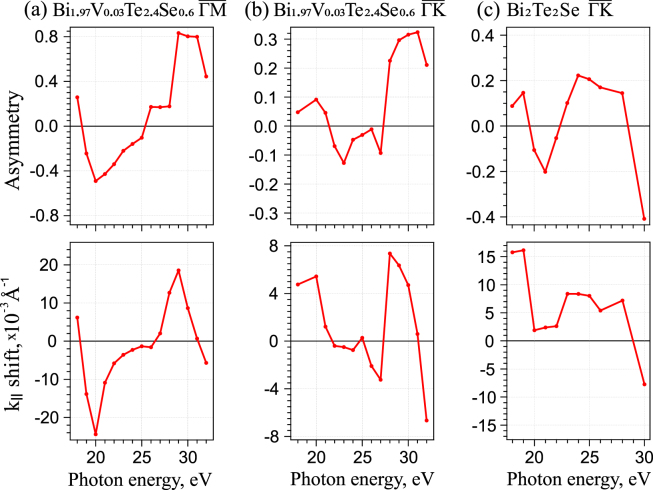
Photon energy dependence of the intensity asymmetry of the DC states with opposite momentum in photoemission spectra (upper line) and the corresponding variation of the magnitude and the sign of the *k*_||_-shifts of the DC states with opposite momenta relative to the bottom CB states (bottom line) measured for Bi_1.97_V_0.03_Te_2.4_Se_0.6_ in the $\overline{{\rm{\Gamma }}{\rm{M}}}$ and $\overline{{\rm{\Gamma }}{\rm{K}}}$ directions –(**a**,**b**) and for Bi_2_Te_2_Se in the $\overline{{\rm{\Gamma }}{\rm{K}}}$ direction– (**c**).

Figure 2 demonstrates in more details the relation between the variation of the TSS intensity asymmetry in photoemission spectra – (upper parts) and the corresponding difference in the *k*_||_-shift of the DC states relative to the non-spin-polarized CB bottom states – (lower parts) for Bi_1.97_V_0.03_Te_24_Se_0.6_ measured in the $\overline{{\rm{\Gamma }}{\rm{M}}}$ and $\overline{{\rm{\Gamma }}{\rm{K}}}$ directions – (a,b) and for Bi_2_Te_2_Se measured in the $\overline{{\rm{\Gamma }}{\rm{K}}}$ direction – (c), respectively, for the photon energy range between 18 and 32 eV. The TSS intensity asymmetry values were estimated in accordance with standard formula:

1 $$A=\frac{{I}_{+{k}_{\parallel }}-{I}_{-{k}_{\parallel }}}{{I}_{+{k}_{\parallel }}+{I}_{-{k}_{\parallel }}},$$

where ${I}_{+{k}_{\parallel }}$ and ${I}_{-{k}_{\parallel }}$ are the intensities of the DC states with opposite momentum. The *k*_||_-shift between the opposite branches of the DC states was estimated as the difference between the *k*_||_-positions of the DC states with opposite momentum relative to the maximum of the bottom CB peak, which were determined from the fitted MDC profiles. One can see that the variation of the sign of the difference in the *k*_||_-shift between the DC states with opposite momentum correlates with the corresponding changes in the sign of the TSS intensity asymmetry. This is most pronouncedly seen for the V-doped TI under measurements in the $\overline{{\rm{M}}{\rm{\Gamma }}{\rm{M}}}$ direction. For the $\overline{{\rm{K}}{\rm{\Gamma }}{\rm{K}}}$ direction and pristine TI such correlation is less visible. However, in any case the inversion of the TSS intensity asymmetry is always followed by corresponding changes in the *k*_||_-shift of the TSS branches. It means that the induced in-plane effective magnetic field is actually generated by SR and can be modified by the changes of the TSS intensity asymmetry under variation of the photon energy. Higher magnitude of the TSS intensity asymmetry observed for the $\overline{{\rm{\Gamma }}{\rm{M}}}$ direction correlates with the results of the theoretical estimations of the photon-energy-dependent polarization of photoemission spectra for the $\overline{{\rm{\Gamma }}{\rm{M}}}$ and $\overline{{\rm{\Gamma }}{\rm{K}}}$ direction^15,25^. Higher asymmetry in Fig. 2 (upper line) is mainly accompanied by higher value of the measured *k*_||_-shift of the DC states, Fig. 2 (bottom line).

Details of difference in behavior of the asymmetry of the TSS intensity and accompanied *k*_||_-shift in different directions of the BZ and different photon energies are determined by the variation of the contributions between *p*_*z*_ and *p*_*x*_, *p*_*y*_ components related to inclusion of the radial and tangential components in spin-orbital texture that influences the TSS PE spectra^25–27^ at different photon energies^25^.

**Figure 3:**
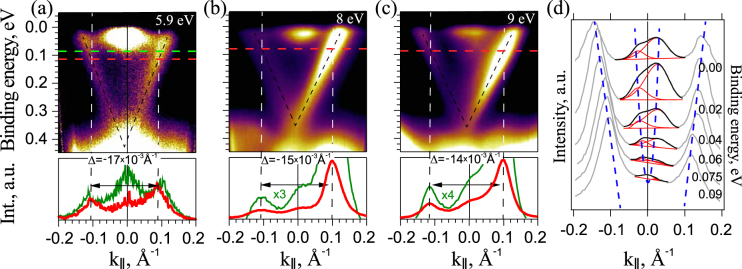
ARPES dispersion map measured for Bi_1.97_V_0.03_Te_2.4_Se_0.6_ under photoexcitation by linearly *p*-polarized laser radiation with photon energy of 5.9 eV in the $\overline{{\rm{\Gamma }}{\rm{K}}}$ direction– (**a**) and SR with photon energy of 8–9 eV in the $\overline{{\rm{\Gamma }}{\rm{M}}}$ and $\overline{{\rm{\Gamma }}{\rm{K}}}$ directions, respectively, –(**b**,**c**). Bottom parts – corresponding MDC profiles of the intensity of the DC states relative to non-spin-polarized CB states measured at the cutting energies (marked by horizontal line) near the CB bottom edge. (**d**) – Series of the MDC profiles for the DC states with opposite momentum and the CB states measured at different BEs between the Fermi level and the CB bottom. Center of the CB peak directly at the CB bottom (lower spectrum) corresponds to the center of the BZ with *k*_||_ = 0.

Analogous effects of correlation between the asymmetry in intensity of the opposite Dirac cone branches and the corresponding *k*_||_-shift of the DC states relative to the non-spin-polarized bottom CB states are observed under photoexcitation by laser and SR with significantly lower photon energy. Figure 3 shows the ARPES dispersion maps measured for V-doped TI Bi_1.97_V_0.03_Te_2.4_Se_0.6_with using *p*-polarized laser (with *hν* = 5.9 eV) – (a) and SR (with *hν* = 8 and 9 eV) – (b), (c), respectively. These dispersion maps are also characterized by asymmetry in the TSS intensity under photoexcitation. In the bottom parts the corresponding intensity profiles of the DC states with opposite momentum are presented relative to the bottom CB state position. The shift of the DP position relative to the bottom CB states is also clearly visible in the presented ARPES dispersion maps. Thus, the TSS intensity asymmetry for these photon energies is also accompanied by corresponding *k*_||_-shift of the DC states and the DP position relative to the bottom CB, which are related to the induced in-plane magnetic field. The direction of the induced magnetic field is determined by the details of the spin-orbital texture contributing at these photon energies.

For the estimation of the *k*_||_-shift of the DC states we have to take into account that the intensity asymmetry in photoexcitation can be also observed for the CB states, too, for instance, due to the final state effect. As a result, when the binding energy (BE) of the CB states is shifted towards the Fermi level from the CB bottom, the distribution of the CB states intensity on *k*_||_ can be also changed. For the dispersion maps presented in Fig. 3 such CB state intensity redistribution can be distinguished. In the insert in Fig. 3(d) the series of the MDC profiles is presented and fitted to show the parabolic-like dispersion and intensity redistribution of the CB states with BE. However, even in this case, if to measure directly at the CB bottom the maximum of the intensity of the CB should correspond to the center of the CB parabolic dispersion. It means that the maximum in intensity directly at the bottom of the CB (or rather centrum of the peak) can be chosen as a good indicator of the $\overline{{\rm{\Gamma }}}$ -point. It means that our estimations of the position of center of the BZ with *k*_||_ = 0 using the MDC profiles just at the CB bottom is not influenced significantly by possible redistribution of the CB state intensity inside the CB.

Thus, from the presented results we can conclude that the asymmetry in the PE intensity of the DC states with opposite momentum is accompanied by the corresponding *k*_||_-shift of the DC position relative to the non-spin-polarized CB states centered at *k*_||_ = 0, which we relate with a generation of effective in-plane magnetic field. The direction and the magnitude of the induced magnetic field vary with photon energy and can be reversed at different photon energies. By increasing the TSS intensity asymmetry the value of the *k*_||_-shift is increasing, too, that should be related to the increased induced in-plane magnetic field.

Below we present the theoretical estimations of the effective magnetic field and magnetization induced by linear *p*-polarized SR. For magnetically-doped TIs with out-of-plane spontaneous easy axis of magnetization (below the Curie temperature) the in-plane effective magnetic field induced by the light (laser or synchrotron radiation) rotates a total magnetization direction towards the in-plane orientation. We consider the simplest case of the light incidence perpendicular to the wave vector (i.e. to the analyzer slit) and, correspondingly, parallel to the spin of the TSS. For our case of analyzer slit parallel to the light incidence one may expect similar behavior. Thus, for magnetically-doped TIs below the Curie temperature two contributions in the total energy of the V-subsystem can be distinguished: one contribution is related to the spontaneous magnetization perpendicular to the surface (easy magnetization axis) and the second one is related to the laser- or SR-generated effective magnetic field determined by the incidence angle of SR (with in-plane and out-of-plane components)^11,14,15^:

2 $${E}_{V}=-\,{K}_{U}{({m}_{z}^{V})}^{2}-({\overrightarrow{m}}^{V}\cdot {\overrightarrow{H}}_{SR}).$$

Here *K*_*U*_ is the constant of uniaxial anisotropy, *m*^*V*^ is the V-subsystem magnetization, ${m}_{z}^{V}$ is the z-axis component of it and ${\overrightarrow{H}}_{SR}$ is the effective field acting on the V impurities from the SR induced magnetization of the electronic subsystem:

3 $${\overrightarrow{H}}_{SR}=\frac{{\tilde{a}}^{2}}{{\mu }_{B}^{2}}{J}_{eV}\overrightarrow{m},$$

where $\tilde{a}=4.24\,\dot{A}$ is the lattice constant for the studied TIs, *J*_*eV*_ ≈ 0.3 eV^22,28,29^ is the exchange constant, which describes s-d interaction between the TSS and the V-ion impurities system and $\overrightarrow{m}$ is the magnetization of the electronic subsystem.

For qualitative estimation of the ${\overrightarrow{H}}_{SR}$ one can use general ideas of the optical orientation theory^30^. Considering that the linearly polarized SR can be decomposed on right and left circularly polarized SR, each of them can depopulate mostly one of the Dirac cone branches^11^, the averaged uncompensated spin accumulation can be represented empirically as $\delta {S}_{x,z}=\frac{\hslash }{2}{\xi }_{x,z}P\tau A$, where *P* is the probability of the electron photoexcitation per unit time, *τ* is the decoherence time of the spins and *ξ*_*x*,*z*_ are the empirical constants. In the simplest case of semiconductor optical orientation ${\xi }_{x}^{0}=\,\sin \,\psi$ and ${\xi }_{z}^{0}=\,\cos \,\psi$^30^, where *ψ* is the angle of incidence. For simplicity, we assume that ${\xi }_{x,z}={\kappa }_{x,z}{\xi }_{x,z}^{0}$, where *κ*_*x*,*z*_ ≈ 1. It should be noted that *Pτ* is the steady-state concentration of the generated holes. Using it, the magnetization of electron sub-system induced by linearly polarized SR can be estimated as ${m}_{x,z}={\mu }_{B}{\kappa }_{x,z}{\xi }_{x,z}^{0}P\tau A$, where *μ*_*B*_ is the Bohr magneton and *A* is the TSS photoexcitation asymmetry (see also^11^). Substituting *m*_*x*,*z*_ into Eq. 3 gives

4 $${\overrightarrow{H}}_{SR}=\frac{{\tilde{a}}^{2}}{{\mu }_{B}}{J}_{eV}P\tau A{({\kappa }_{x}\sin \psi \mathrm{,0,}{\kappa }_{z}\cos \psi )}^{T}\mathrm{.}$$

Using it one can rewrite the energy of V-subsystem in spherical coordinates:

5 $$\begin{array}{rcl}{E}_{V} & = & -{K}_{U}{m}_{V}^{2}\,\cos \,{}^{2}\theta -{m}_{V}{H}_{SR}{\kappa }_{x}\,\sin \,\psi \,\sin \,\theta \,\cos \,\phi \\  &  & -{m}_{V}{H}_{SR}{\kappa }_{z}\,\cos \,\psi \,\cos \,\theta ,\end{array}$$

where ${m}_{V}=|{\overrightarrow{m}}^{V}|$, ${H}_{SR}=\frac{{\tilde{a}}^{2}}{{\mu }_{B}}{J}_{eV}P\tau A$ and (*ϕ*, *θ*) are the spherical angles of the V-subsystem magnetization. Minimization of this energy gives the following equilibrium position:

6 $$\sin \,\phi \,=\,0,$$

7 $${H}_{SR}\,\sin \,(\theta -\eta )+{K}_{U}{m}_{V}\,\sin \,(2\theta )=\mathrm{0,}$$

where *η* is slightly differs from *ψ* and $\sin \,\eta ={\kappa }_{x}\,\sin \,\psi /\sqrt{{\kappa }_{x}^{2}\,{\sin }^{2}\psi +{\kappa }_{z}^{2}\,{\cos }^{2}\psi }$. Hence *η* = *ψ* in case of *κ*_*x*_ = *κ*_*z*_. Eq. 6 demonstrates that in plane projection of V magnetization lies in the plane of SR incidence, at the same time Eq. 7 describes V magnetization direction in this plane. Two special cases easily follow from this equation: in case of SR absence *H*_*SR*_ = 0 therefore V magnetization is perpendicular to the surface, and in case of *T* > *T*_*C*_ there is no anisotropy (*K*_*U*_ = 0) therefore V magnetization lies along the direction *η*, defined by the SR wave vector. To find the module of V-subsystem magnetization *m*_*V*_ in general case let us consider that V impurities do not interact directly to each other. In this case our system is the system of non interacting spins in external effective field, which is the vector sum of the SR induced effective field and the anisotropy field. Hence *m*_*V*_ is represented by Brillouin function:

8 $${m}_{V}=g{\mu }_{B}SN{B}_{S}(\frac{g{\mu }_{B}S\sqrt{{H}_{SR}^{2}+4{K}_{U}^{2}{m}_{V}^{2}+4{H}_{SR}{K}_{U}{m}_{V}\,\cos \,\psi }}{T}),$$

where *g* ≈ 2 is the g-factor, *S* = 3/2 is the spin of V ion and *N* = 8 × 10^12^ cm^−2^ is the impurity concentration. The Akulov-Zener classical theory of the temperature dependence of ferromagnetic anisotropy energy predicts ${K}_{n}(T)/{K}_{n}(0)={({M}_{U}(T)/{M}_{U}(0))}^{\frac{n(n+1)}{2}}$, where *K*_*n*_ is the n-th order anisotropy constant. In the case of uniaxial anisotropy and the magnetization approximated as *M*(*T*) ≈ *M*(0)(1 − *T*/*T*_*C*_)^1/3^, the anisotropy constant can be estimated as *K*_*U*_ = *K*_*U*_(0)(1 − *T*/*T*_*C*_), where ${K}_{U}(0)\approx {\tilde{K}}_{U}/{g}^{2}{\mu }_{B}^{2}{S}^{2}N$ and ${\tilde{K}}_{U}\approx 6$ meV is the anisotropy energy per impurity^31^. After solving Eq. 8 the magnetization of V-subsystem can be calculated as ${\overrightarrow{m}}^{V}={m}_{V}{(\sin \theta \mathrm{,0,}\cos \theta )}^{T}$. In the case of *T* > *T*_*C*_ anisotropy disappear (*K*_*U*_ = 0), therefore Eq. 8 can be simplified to:

9 $${\overrightarrow{m}}^{V}=g{\mu }_{B}SN{B}_{S}(g{\mu }_{B}S{H}_{SR}/T){(\sin \psi \mathrm{,0,}\cos \psi )}^{T}\mathrm{.}$$

**Figure 4:**
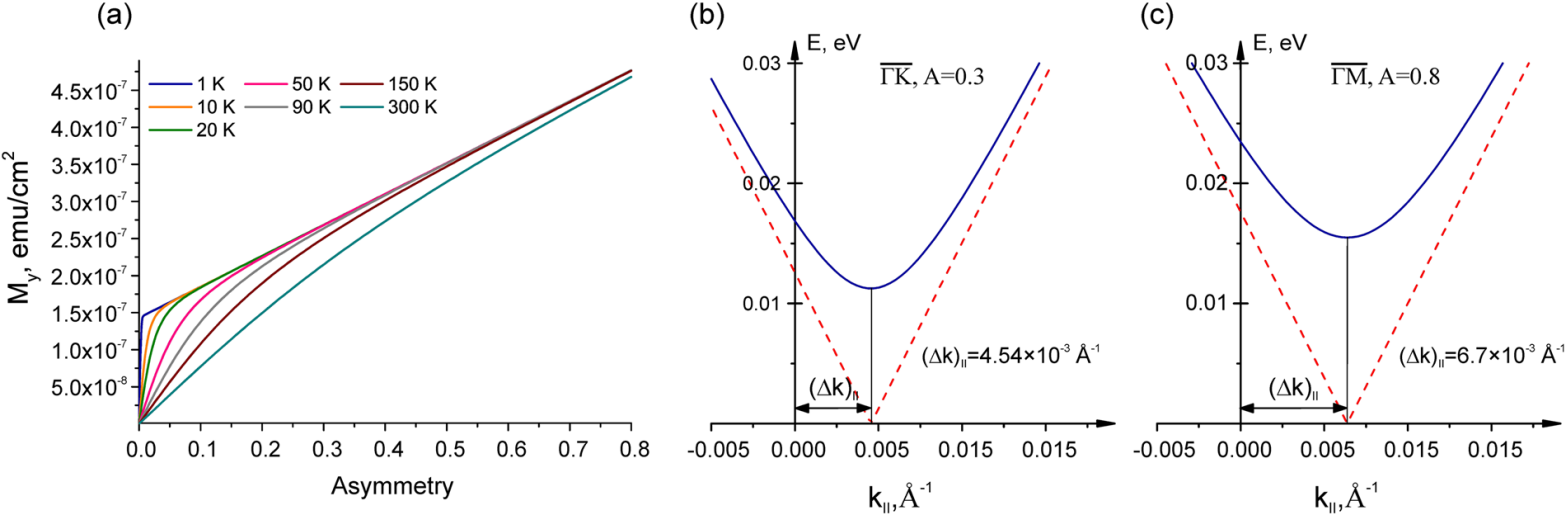
(**a**) –Calculated dependence of the in-plane component of the SR-induced magnetization as a function of the asymmetry in the intensity of the DC states with opposite momentum. (**b**,**c**) –Calculated modification of the dispersion of the upper DC states (the *k*_||_-shift) due to the induced in-plane magnetic field related to the experimentally observed TSS intensity asymmetries (A equals 0.3 and 0.8, respectively). The conduction and valence band states are not shown. Dashed red and solid blue lines depict the upper DC without and with the opening of an energy gap (see text).

The estimations prove that the anisotropy term is significantly smaller than the SR term. Therefore this equation is valid with a good accuracy for all temperature range. The calculated dependence of the in-plane component of the total magnetization $\vec{M}=\vec{m}+{\vec{m}}^{V}$ (under experimental conditions *ψ* = 50° and *Pτ* ≈ 3.5 × 10^13^ cm^−2^) on the in-plane asymmetry value (*A*) for different temperatures is represented on Fig. 4(a).

It means that the magnitude of the induced in-plane magnetization can be estimated (for the used experimental conditions) directly from these dependences for different experimental values of the TSS intensity asymmetries. For temperature of 20 K and the averaged TSS intensity asymmetry of about 0.3 the magnitude of the induced magnetization is on the level of 2.7 × 10^−7^ emu cm^−2^ (see from Fig. 4(a)). For the TSS intensity asymmetry of about 0.8, which is the highest one observed experimentally, the induced magnetization should reach the value of about 4.8 × 10^−7^ emu cm^−2^.

The complete electron Hamiltonian of the considered system including both electron-electron and electron-vanadium interactions in the mean field approximation can be written as:

10 $${\hat{H}}_{e}=\hslash {V}_{D}[\overrightarrow{k}\times \overrightarrow{\sigma }]{\overrightarrow{e}}_{z}+\frac{{\tilde{a}}^{2}}{{\mu }_{B}}{J}_{eV}({\overrightarrow{m}}^{V}\cdot \overrightarrow{\sigma })+\frac{{\tilde{a}}^{2}}{{\mu }_{B}}U(\overrightarrow{m}\cdot \overrightarrow{\sigma }),$$

where ${V}_{D} \sim (3.7-4.2)\times {10}^{7}\,cm/s$ is the velocity taken from the TSS dispersion law, $\overrightarrow{\sigma }$ is the vector of the Pauli matrices and *U* ≈ 0.2 eV is the Hubbard parameter. The energy spectrum in this case has the following form:

11 $$E=\pm \sqrt{{\hslash }^{2}{V}_{D}^{2}|\overrightarrow{k}{|}^{2}+{{\rm{\Delta }}}_{y}^{2}+{{\rm{\Delta }}}_{z}^{2}-2\hslash {V}_{D}{k}_{x}{{\rm{\Delta }}}_{y}},$$

where ${{\rm{\Delta }}}_{y}=\frac{{\tilde{a}}^{2}}{{\mu }_{B}}{J}_{eV}{m}_{y}^{V}+\frac{{\tilde{a}}^{2}}{{\mu }_{B}}U{m}_{y}$ and ${{\rm{\Delta }}}_{z}=\frac{{\tilde{a}}^{2}}{{\mu }_{B}}{J}_{eV}{m}_{z}^{V}+\frac{{\tilde{a}}^{2}}{{\mu }_{B}}U{m}_{z}$. Taking this into account one can write the resulting *k*_||_-shift as:

12 $${({\rm{\Delta }}k)}_{\parallel }=(\frac{{\tilde{a}}^{2}}{{\mu }_{B}}{J}_{eV}{m}_{y}^{V}+\frac{{\tilde{a}}^{2}}{{\mu }_{B}}U{m}_{y})/\hslash {V}_{D}\mathrm{.}$$

The resulting band structure modification under influence of the induced out-of-plane and in-plane magnetization calculated for the used experimental conditions (see above) is shown in Fig. 4(b,c). The induced in-plane magnetization emerges in the *k*_||_-shift of the DC in the direction orthogonal to the induced magnetic field (or magnetization).

Because the laser or SR with an oblique incidence angle generates both the in-plane and out-of-plane components of the induced magnetic field it should be followed by the opening of an energy gap at the DP due to time-reversal symmetry breaking. However, in the current work we do not analyze the energy splitting of the DC states at the DP. While the opening of a gap at the Dirac point is also shown in Fig. 4(b,c). At the same time, Fig. 4(b,c) show that the theoretically estimated values of the *k*_||_-shift of the DP position due to the in-plane component of the magnetic field for the averaged experimental asymmetries (*A* = 0.3) should be on the level of about 4–5 × 10^−3^ Å^−1^. It correlates well with the experimentally observed *k*_||_-shifts presented in Fig. 2. Here it is necessary to note that the *k*_||_-shift of the DP relative to the $\overline{{\rm{\Gamma }}}$ -point should be twice smaller than the *k*_||_-shift between the opposite DC branches determined relative to the position *k*_||_ = 0 determined using the bottom CB states ((*k*_1_ − *k*_0_) – (*k*_2_ − *k*_0_)). In the case of the higher TSS intensity asymmetry (till 0.8) the *k*_||_-shift between the opposite branches of the DC states can reach experimentally the value of about 15–18 × 10^−3^ Å^−1^. It corresponds to the *k*_||_-shift of the DP relative to the $\overline{{\rm{\Gamma }}}$ -point of about 7–9 × 10^−3^ Å^−1^. In the case of theoretical estimation it reaches the value of about 7 × 10^−3^ Å^−1^.

Thus, at temperatures below the Curie temperature the rotation of the magnetization takes place from the spontaneous out-of-plane magnetization towards the in-plane magnetization generated by the laser or SR. This reorientation is restricted by the photonic beam size at positions that can be scanned along the surface with consequent controlled locally-restricted remagnetization with possible local modification of the DC gap (which can be measured, for instance, by corresponding changes of magnetoresistivity) that can be effectively used in spintronics. The direction and the magnitude of the induced magnetization can be modified by changing the photon energy. For temperatures above the Curie temperature a spontaneous out-of-plane magnetization is zero and only the induced in-plane magnetization is present under the laser or SR excitation. The induced magnetization has a surface 2D character.

## Conclusions

We have shown a correlation between the intensity asymmetry of the Dirac cone states with opposite momentum under photoexcitation and the *k*_||_-shift of the DC states (and the DP position) observed in the ARPES dispersion maps for V-doped and pristine TIs with the photon energy variation. Theoretical analysis of the induced magnetic field due to the hole spin accumulation under photoexcitation and the estimation of the resulting *k*_||_-shift of the DC states caused by the induced in-plane magnetic field correlate well with the experimental data. Therefore, we can conclude that the asymmetry in photoexcitation of the DC states with opposite momentum is accompanied by laser- or SR-generated in-plane magnetic field (and magnetic ordering) which is responsible for the observed *k*_||_-shift of the DP position. Our findings open new perspectives for effective manipulation by the local magnetization restricted by the laser or SR beam spot which can be scanned along the surface. At the same time this effect should be taken into account in analysis of electronic and spin structure in angle-resolved photoemission measurements.

## Methods

The ARPES measurements of the DC states with *p*-polarized SR shown in Figs 1 and 3(b,c) were carried out at the BaDElPh beamline^32^ at Elettra (Trieste, Italy) in the direction along the SR incidence plane both for the $\overline{{\rm{\Gamma }}{\rm{K}}}$ and $\overline{{\rm{\Gamma }}{\rm{M}}}$ direction of the BZ using a SPECS Phoibos 150 analyzer. The incidence angle of SR for these experiments was 50° relative to the surface normal. The laser experiment (Fig. 3(a)) was carried out in ISSP at Tokyo University (Japan). The laser pulse was linearly *p*-polarized with photon energy of 5.9 eV. The laser beam incidence angle was 45° relative to the surface normal. Part of work was carried out in the resource center “Physical methods of surface investigation” (PMSI) of the Research park of Saint Petersburg State University.

The single crystals of pristine TI Bi_2_Te_2_Se and magnetically-doped TI Bi_1.97_V_0.03_Te_2.4_Se_0.6_ were synthesized by using a modified vertical Bridgman method. Clean surfaces of the TIs were obtained by a cleavage in ultrahigh vacuum. The base pressure during the experiments was better than 1 × 10^−10^ mbar.
